# Nourishing the Mind: How Food Security Influences Mental Wellbeing

**DOI:** 10.3390/nu16040501

**Published:** 2024-02-09

**Authors:** Ovinuchi Ejiohuo, Helen Onyeaka, Kingsley C. Unegbu, Obinna G. Chikezie, Omowale A. Odeyemi, Adebola Lawal, Olumide A. Odeyemi

**Affiliations:** 1 Department of Psychiatric Genetics, Poznan University of Medical Sciences, 60-806 Poznan, Poland; 2 Doctoral School, Poznan University of Medical Sciences, Bukowska 70, 60-812 Poznan, Poland; 3 School of Chemical Engineering, University of Birmingham, Edgbaston, Birmingham B15 2TT, UK; 4 Department of Vegetable Crops, Poznan University of Life Sciences, 60-594 Poznan, Poland; k.unegbu@gmail.com; 5 Department of Seed Science and Technology, Poznan University of Life Sciences, 62-081 Poznan, Poland; gabrielchikezie@gmail.com; 6 College of Nursing, Obafemi Awolowo University Teaching Hospital Complex, Ile Ife 220005, Osun, Nigeria; oaodeyemi@gmail.com; 7 Hospital Management Board, Government House and Protocol Clinic, Akure 340283, Ondo, Nigeria; pharmadebola@gmail.com; 8 Office of Research Services, Research Division, University of Tasmania, Hobart, TAS 7001, Australia; olumide.odeyemi@utas.edu.au

**Keywords:** food security, food insecurity, mental health, stress, anxiety, depression, nutrition, hygiene, wellbeing

## Abstract

Food insecurity is a significant public health problem worldwide and critical to mental health. There is a complex relationship between food security and mental health. We carried out a narrative review study aiming to address how food insecurity impacts mental wellbeing by focusing on the mental health repercussions of food insecurity, recognizing its pivotal role in attaining Sustainable Development Goals 2 (on hunger) and 3 (on enhancing global wellbeing). A comprehensive search was conducted on PubMed and Google Scholar, incorporating Google searches for pertinent reports and policy documents. To address these questions, we emphasized and elucidated the interconnectedness between food security and mental health. The review shows that food security and mental health share a profound relationship influenced by multifaceted factors like socioeconomic conditions, access to nutritious food, and societal inequalities. We then provide recommendations for integrating food security into mental health strategies based on the insights and conclusions drawn. Strategies ranging from sustainable farming practices to urban agriculture initiatives and digital mental health services demonstrate avenues for enhancing food safety and mental wellbeing. This highlights the need for collaborative interdisciplinary efforts and systemic reforms to address these interconnected challenges.

## 1. Introduction

As of 2020, one in nine persons worldwide suffers from chronic hunger due to food insecurity [1]. This means that global efforts to eradicate hunger and food insecurity by 2030, in line with the Sustainable Development Goals (SDGs), might be slipping further behind schedule. Food security is realized when individuals consistently have the physical and economic means to access sufficient safe and nutritious food that aligns with their dietary preferences, supporting a healthy and active lifestyle [2]. The absence of these is considered food insecurity. According to the 2023 report by the Food and Agricultural Organization of the United Nations (FAO), there was a 122 million increase in global hunger from 2019 to 2022. There was also a 20% increase in food insecurity (an inability to satisfy one’s food demand in the future) from 2021 to 2022 [1]. Global hunger, impacted by conflict, climate change, increasing food prices, and inequalities, may no longer be rising. However, it is still above pre-pandemic levels (815 million people were affected in 2017 [1] and 333 million people were affected in 2023 [3]. We might attribute these positives to interventions during and after the pandemic, but the war in Ukraine, which has impacted the global grain trade, has contributed to its slow decline [1]. With the war on Gaza, which started on 7 October 2023, there could even be a re-emergence of an upward trend in global food insecurity or a worsening of the situation. Beyond local food insecurity, conflicts impact global food systems, agricultural production, and supply chains [4] by reducing production output, compromising supply chains, hindering access to crucial resources like fertilizers and agrichemicals, and escalating prices, thus posing significant challenges to food security worldwide [5,6].

Food security significantly impacts mental health outcomes, influencing stress levels, emotional stability, cognitive function, and general psychological wellbeing [7]. A review by Myers (2020) affirms the correlation between food security and psychological distress and critical areas for further insight, such as psychological indicators (eating disorders and suicide), contextual factors (environmental and personal factors), racial inequality, and food scarcity [8]. Women are particularly negatively impacted by food insecurity [1]. In places devastated by war, women have increased susceptibility to psychological stress, which affects the wellbeing of both mother and child [9].

**Figure 1:**
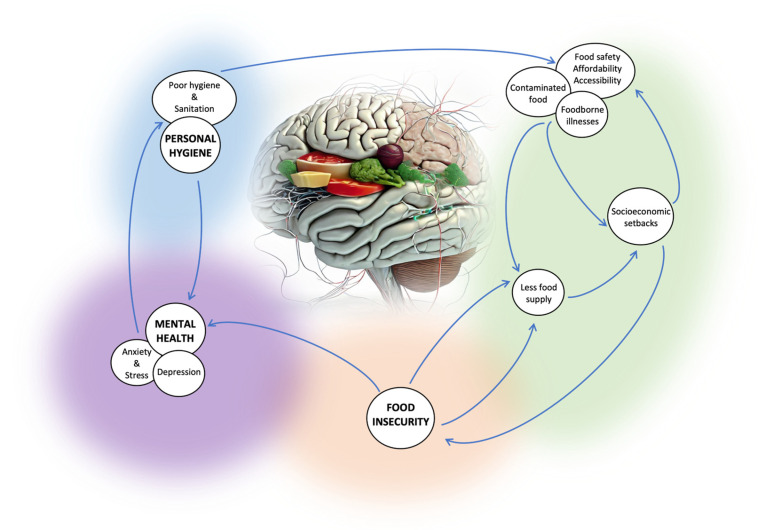
Complex relationship between hygiene, food security, and mental health.

There is a complex relationship between food security, hygiene, and mental health, as shown in Figure 1. Food safety and accessibility can be impacted by poor hygiene standards, which can lead to food insecurity. Poor hygiene and sanitation can result in foodborne illnesses (such as gastroenteritis, Hepatitis A, salmonellosis, listeriosis, and food poisoning) contaminated with pathogenic microorganisms such as *Escherichia coli*, *Staphylococcus aureus*, *Listeria monocytogenes*, *Salmonella* spp., and Hepatitis A virus [10]. This leads to food waste and financial setbacks. When food becomes contaminated, it creates a situation of lesser food supply. The study by Vuong et al., 2022 found independent correlations between lower physical and mental component scores (PCS and MCS) and household food insecurity, as well as inadequate water usage, highlighting a connection between hygiene, food insecurity, and overall health [11]. Poor hygiene in communities limits access to clean water, proper storage, and hygiene education, impacting food safety and availability and contributing to food insecurity [12]. In the study by Mshida et al., 2018, undernutrition among young children was linked to poor water, sanitation, and hygiene (WASH) practices and various sociocultural factors such as early complementary food introduction and consumption of un-boiled cow milk [13]. Consequently, inadequate food access can cause anxiety and stress, which can harm one’s mental health. Conversely, poor mental health can have an impact on personal hygiene and food security. The study by Stewart et al., 2022 highlights the impact of declining personal hygiene on depression, emphasizing its influence on various life aspects and the need for intervention [14].

These interconnected factors emphasize the importance of addressing hygiene, food security, and mental health collectively for overall wellbeing. The intricate relationship between food security and mental health also highlights the urgent necessity for comprehensive research and interventions targeting both areas to alleviate their extensive implications. Thus, this review study primarily concentrates on the mental health repercussions of food insecurity, recognizing its pivotal role in attaining Sustainable Development Goals 2 (hunger) and 3 (health and wellbeing) and enhancing global wellbeing.

Therefore, our narrative review aims to address the question of how food security impacts mental wellbeing alongside its pivotal role in achieving sustainable development. Based on this, we postulate the following questions:

- Does food security significantly impact mental health outcomes, and how does this relationship contribute to achieving Sustainable Development Goals related to hunger and health?
- To what extent do socioeconomic factors influence the interconnectedness between food security and mental health?
- How can comprehensive interventions that involve food security and mental health address these multifaceted implications for global wellbeing?

To address these questions, we emphasize and elucidate the interconnectedness between food security and mental health. Based on the insights and conclusions drawn, we then provide recommendations for integrating food security and mental health strategies that will effectively achieve global wellbeing.

## 2. Methodology

A comprehensive search was conducted on PubMed and Google Scholar until January 2024, incorporating Google searches for pertinent reports and policy documents. A systematic query using key terms such as ‘food security’, ‘food insecurity’, ‘mental health’, and ‘mental wellbeing’ was carried out.

The inclusion criteria encompassed studies on food security, food insecurity, mental health, factors influencing either or both food security and mental health, interventions, policy briefs, reports, and Sustainable Development Goals on hunger and wellbeing as they relate to the topic of our study.

Our review considered various sources such as systemic reviews, conference papers, meta-analyses, observational studies, clinical trials, policy documents, reports, relevant webpages, blog posts, and published books/sections.

The selection process involved more than just screening titles and abstracts, and the analysis of these diverse studies informed the narrative review’s insights, conclusions, and recommendations.

## 3. Defining Food Security and Mental Health

Drawing from the Food and Agricultural Organization of the United Nations (FAO)’s four dimensions of food security, this term can be defined by the availability, accessibility, nutrient utilization, and sustainability of food over time; food security ensures not only the presence of food but also its consistent access, practical use, and protection against potential threats that could undermine nutritional wellbeing [15]. According to the World Health Organization (WHO), mental health refers to a condition of mental wellness that allows individuals to manage stress, utilize their skills effectively, excel in learning and work, and actively participate in their community [16]. Having adequate access to safe and nutritious food can enhance mental wellbeing by alleviating the stress and anxiety associated with food insecurity. In the study by Chayama et al., 2023, enhanced emotional wellbeing and decreased stress, worry, and anxiety in people living with human immunodeficiency virus (PLHIV) were linked to increased availability of adequate and nutritious food alongside four other contributing factors (reduced financial difficulty, social support, fewer eating barriers, and improved self-control and self-esteem) [17]. In another study by Wolfson et al., 2021 [18], low-income Americans’ mental health suffered as a result of the COVID-19 pandemic, especially for those who are facing food insecurity. Over one-third of them experienced psychological distress early in the pandemic, primarily anxiety, despair, and high levels of stress; higher levels of distress were correlated with poorer levels of food insecurity [18]. The study by Mora et al. in 2022 found that the COVID-19 pandemic had a notable impact on farmworkers’ mental health and significantly increased food insecurity to 37%, being influenced by factors like experiencing COVID-19 symptoms and demographics such as having children, foreign-born status, and educational level linked to food insecurity [19].

It is essential to look beyond economic accessibility when considering food insecurity. The study by Vaudin et al., 2022, showed that solely assessing economic access may overlook those requiring food assistance. They found that older adults facing both economic food insecurity and physical difficulties accessing food had notably lower diet quality and higher depression scores compared to those in economically secure households [20]. The study by Long et al. in 2022 revealed a concerning trend of declining dietary quality among older adults in the US from 2001 to 2018. Over half of older adults maintained poor dietary quality [21]. This decline may signal challenges in accessing healthier food options, which are linked to limited physical access to nutritious food. This situation is even more dire when considering low-income older adults, as highlighted in the study by Qin et al., 2022, where poor nutrient intake and dietary quality were observed in low-income older adults older than sixty in the United States [22]. Similarly, Selvamani and Elgar in 2022 found that among middle-aged and older persons in India, food insecurity emerged as a significant social determinant associated with several measures of poor health and wellbeing [23]. The same can be said for people with disabilities. In the study by Engelman et al. in 2020, worries about food shortages, compounded by concerns about COVID-19 and social isolation, were found to intersect to impact food insecurity among deaf and hard-of-hearing individuals, highlighting the intricate relationship between food accessibility, health-related worries, and mental wellbeing in this population [24].

The availability of enough and nourishing food is influenced by a variety of socioeconomic factors that are intertwined with food security. These factors include education, healthcare access, income, housing, employment, government policy, and social support [25,26,27]. The existence or nonexistence of these components can have a substantial effect on a person’s mental health, influencing their capacity to manage stress, preserve emotional stability, and successfully negotiate obstacles related to food insecurity.

Living in places considered “food deserts” where there is poor access to supermarkets or fresh vegetables might exacerbate food insecurity. “Food deserts” are areas and communities with little availability of reasonably priced and nutritious food [28]. For instance, there is an increased risk of obesity, as unhealthy eating habits are promoted due to less healthy choices of food. In the study by Brace et al. in 2020, the limited presence and low involvement in food assistance programs and the limited operating hours of farmer’s markets in Hawaii were shown to contribute to the state’s obesity crisis by preventing citizens from having easy access to fresh produce [29]. Marginalized communities are particularly affected by food deserts. In a United States study by Samson and Hannibal in 2021, when choosing food sources, minority communities gave more weight to aspects like affordability, ease of transit, food variety, and availability of organic options. In addition, these communities must contend with substantially longer travel times than white populations [30].

**Figure 2:**
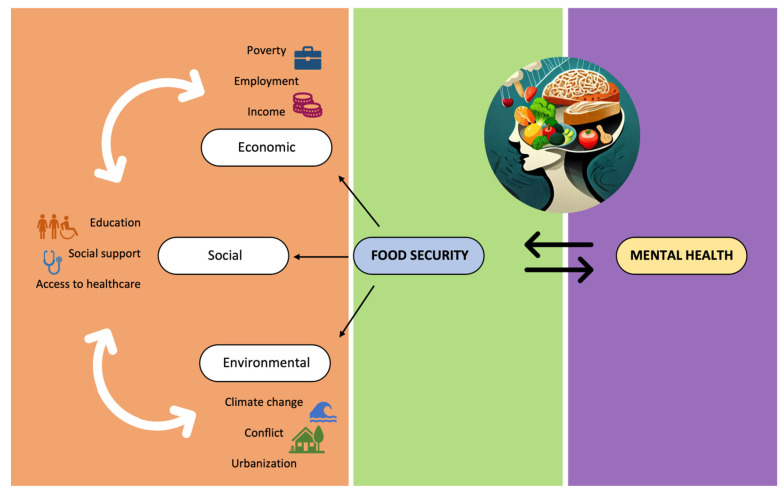
Socioeconomic factors influencing food security and mental health.

Unstable living arrangements or subpar housing can exacerbate the stress associated with accessing food, which can have an adverse effect on mental health. In the study by Carrere et al. in 2022, people who had housing insecurity were more likely to have poor mental health, which was made worse by the cohabitation of other life concerns such as inaccessibility or affordability of good food [31]. This shows how food security, housing security, and mental health can become an unending loop. This can further be worsened in people with existing mental illness. Figure 2 shows the interplay between socioeconomic factors that influence mental health outcomes in populations experiencing food insecurity.

## 4. Impact of Socioeconomic Status on Food Security and Mental Health

The availability of nutritious food is often associated with higher income and steady employment. Financially stable people can purchase nutritious foods, lessening the stress of food insecurity. On the other hand, a lack of money can exacerbate anxiety and negatively damage mental health, especially if it makes it difficult to afford enough food [18,32]. This can also be connected to education, as the more financially stable one is, the more easily one can access quality education. A lack of education may impede access to resources that support food security and lead to limited awareness of nutritional needs, which may harm mental health through stress and ambiguity. A study by Henry in 2017 involving food-insecure students showed that the students commonly experience stigma and shame, hindering their willingness to seek support from both family and government aid programs [33]. Therefore, isolation or a lack of support during a period of food shortage may exacerbate mental health difficulties, and having a supportive network can help reduce some of the stress associated with food insecurity. In the study by Na et al. in 2018, among individuals most impacted by food insecurity in sub-Saharan Africa, social support improved the causal relationship between food security and mental health [34]. People with mental health issues also may find it difficult to navigate complex health systems to meet their nutritional needs and access adequate care. Schwarz et al. in 2022 identified barriers such as poor patient pathways, fragmented care, communication issues, regional disparities, limited consultation time, and patients’ challenges in recognizing needs and accessing healthcare due to socioeconomic factors [35].

## 5. The Psychological Toll of Food Insecurity

**Figure 3:**
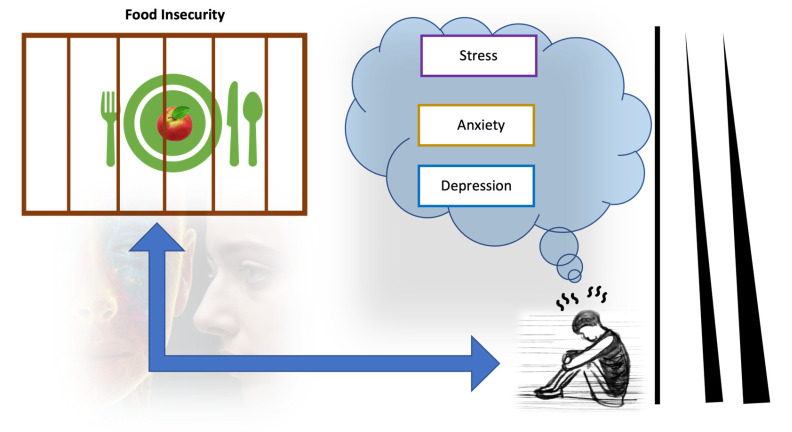
Illustration of the psychological toll of food insecurity.

Food insecurity is considered a psychosocial stressor that negatively impacts mental health (Figure 3), leading to increased levels of psychological disorders such as anxiety, depression, shame, and stress [36,37]. In the study by Wolfson et al., 2021, compared to low-income adults with high food security who screened at 14.3% for depression, 20.5% for anxiety, and 17.8% for high perceived stress, low-income adults with very low food security screened at 54.9% for depression, 58.9% for anxiety, and 66.3% for high perceived stress [18]. The importance of food security as a health predictor often outweighs the impact of money. This is highlighted by the substantial correlation between reduced food security and higher risks and a higher number of chronic diseases [38]. For instance, stroke is a chronic illness that affects the brain and triggers mental conditions such as stress, mood disorders, depression, anxiety, and sleep disturbances [39,40,41].

Stress can result from not having access to enough food because of the uncertainty and anxiety surrounding one’s basic need for nourishment. This stress results from anxieties about where one will get their next meal, which causes emotional strain and anxiety about the availability and quality of food and one’s own and one’s family’s wellbeing. Insufficient protein can affect mood, cognition, and energy levels due to its impact on neurotransmitter production and nutrient deficiencies like low iron, leading to mood swings, cognitive decline, fatigue, and irritability [42,43,44].

Food insecurity and poor nutrition quality can weaken the immune system, impacting the variety and balance of the gut microbiota. Stress changes the composition of the gut microbiota and may cause dysbiosis (an imbalance of the gut microbiome) [45,46]. Consuming a fiber-rich diet promotes the growth of gut bacteria that produce butyrate [47,48,49], consequently lowering stress levels and depression [50]. Zhang et al.’s 2023 study observed that in individuals with high dietary fiber intake, there was no association between symptoms of depression and dietary inflammatory index (DII), a sociodemographic characteristic representing dietary inflammation and C-reactive protein (CRP) [51]. A previous study by Caspani et al., 2019 explored the mechanism used by the gut microbiota in modulating metabolites that influenced mood changes in major depressive disorder (MDD) through actions in the gut–brain axis [52]. The gut–brain axis affects stress responses, emotional states, and regulatory systems such as the hypothalamus–pituitary–adrenocortical (HPA) axis. It involves interactions between the gut bacteria and neurological function [53].

Children who experience food insecurity are more vulnerable to adverse effects on their mental health, development, and overall wellbeing [54]. In the study by Ling et al. in 2022, [55] both adult and child food insecurity significantly correlated with higher stress, anxiety, and depression in parents and fear in their children after accounting for demographic factors. In the study, both adult and child food insecurity negatively impacted parental stress, anxiety, depression, and children’s fear, while parental depression was linked to child food insecurity. This emphasizes the critical need to enhance food security in low-income families to improve mental wellbeing for both parents and young people [55]. Food insecurity is associated with lower self-esteem, particularly in children from impoverished families. In the study by Godrich et al. in 2019, food-insecure children exhibited lower self-esteem and a reduced ability to make healthy choices compared to those from food-secure households, notably impacting girls more than boys [56]. In a recent study by Bell et al. in 2023, food insecurity negatively impacted children’s overall wellbeing, influencing their understanding of limited resources and their role in supporting their families [57]. When interventions and policies reduce food insecurity in adolescents and children, there is an increase in positive childhood experiences (PCEs), as shown in the study by Zhang et al. in 2023, wherein mild and moderate/severe food insecurity was associated with lower rates of positive child experiences across different age groups [58].

To better understand how food security affects young people’s mental development and overall mental health, it is crucial to examine food’s role in children’s development. Nutrition is important for children’s mental development because the brain needs nutrients to develop properly. A child’s early nutrition shapes brain development, supplying vital nutrients for optimal cognitive growth and better interaction with their environment. Undernourished children may not fully develop cognitive, physical, and socioemotional skills [59]. Nutrition is a critical factor in early fetal brain development [60,61]. For children’s cognitive function, memory, attention, and emotional regulation, among other things, nutrients including iron; zinc, iodine; omega-3 fatty acids; and vitamins B, C, D, and E are crucial [62]. The study by Nyaradi et al. in 2013 [63] noted that these micronutrients notably impact children’s cognitive development. An unhealthy diet, therefore, can result in deficiencies and imbalances in micro- and macronutrients [63]. In a study by Roberts et al. in 2022, malnourished children who were given micronutrient supplements had significantly improved cognitive performance [62].

As we have seen so far, food security has a profound impact on youth and adolescent mental health. Below, we present three case studies: war in Palestine (since it is the most recent conflict), the Democratic Republic of Congo, and Afghanistan and Yemen (since they are the top three countries most affected by hunger) [64].

### 5.1. Case Study 1: War in Palestine

Gaza’s children face critical food insecurity, resulting in poor diets and widespread nutritional deficiencies. According to the World Food Programme (WFP), food insecurity among Palestinian children is a pressing issue, with 59.4% affected in Gaza and 63% affected in the West Bank, worsened by prolonged conflict, political divisions, economic instability, and restricted access to resources. Generally, around 2.2 million individuals, including 576,600 experiencing catastrophic hunger, are in acute food insecurity within the Gaza region, out of the total 5.3 million population across the State of Palestine [65].

According to the research of Hammoudeh et al. (2022), [66] in conflict environments such as the occupied Palestinian territories (oPt), food security is a crucial socioeconomic factor of mental health. Objective economic metrics (household food consumption) had less of an impact on mental health than subjective ones, especially regarding the feeling of being economically disadvantaged, thus emphasizing the crucial role of adequate and anxiety-free access to food in conflict settings. This finding elevates food security to a critical factor in mental health [66].

### 5.2. Case Study 2: Democratic Republic of Congo

The effect of food insecurity on children in the Democratic Republic of the Congo is severe, with an estimated 900,000 children suffering from severe malnutrition among a total of 25.4 million acutely food-insecure individuals identified between July and December 2023. Of these 25.4 million people, 6.9 million were internally displaced individuals experiencing acute food insecurity from July to December 2023, with 4.4 million children, pregnant, and breastfeeding women facing acute malnutrition [67]. Over 25 years of conflict and displacement have left 26 million severely hungry, leading to resilience tests through climate shocks, Ebola outbreaks, and the economic impacts of COVID-19 [64].

### 5.3. Case Study 3: Afghanistan and Yemen

In Afghanistan, four decades of conflict worsened by the 2021 government collapse, Taliban takeover, and economic decline have left 19.9 million severely hungry, including 4 million malnourished women and children. An eight-year civil war in Yemen has also led to 17 million facing severe hunger, pushing nutritious food out of reach and escalating malnutrition rates among women and children [64]. These crises fuel instability, displacement, and poverty, severely impacting mental health due to hunger and related challenges.

## 6. Vulnerable People, Food Security, and Mental Health

The role of diets in neurological function is often likened to a double-edged sword. For instance, the Western diet, which is characterized by high levels of saturated fat, refined sugar, and processed foods, has been associated with impaired learning and memory. Conversely, certain dietary aspects like polyphenols and dietary patterns like the Mediterranean diet exhibit antioxidant and anti-inflammatory properties [68]. Interestingly, diet significantly influences the gut microbiota, which, in turn plays a vital role in overall health [69]. Dietary fibers are essential for gut microbiota health, promoting the production of beneficial by-products like short-chain fatty acids (SCFAs), in particular, acetate, propionate, and butyrate [70,71].

Neurological conditions such as stroke, dementia, Parkinson’s disease, and autism spectrum disorders often lead to increased vulnerability to nutritional deficiencies, gastrointestinal issues, and nutritional problems [72]. For example, recent research by Kang et al. in 2024 identified the gut–brain communication pathway [73]. The team highlighted how enteric bacteria modulate host behavior and potentially impact neurological health by revealing that vitamin B12 reduces cholinergic signaling in the nervous system by altering the methionine/S-adenosylmethionine cycle in the intestine. This metabolic interaction between these pathways affects cholinergic signaling by limiting the availability of free choline necessary for neurons to synthesize acetylcholine [73]. In another case–control study involving 148 Danish multiple sclerosis cases and 148 matched healthy controls, significant differences were found in 61 bacterial species between multiple sclerosis cases and healthy controls. The study highlighted notable disparities in the gut microbiota of multiple sclerosis patients directly correlated with blood biomarkers of inflammation [74]. This underscores the intricate relationship between diet, gut microbiota, and the profound impact on neurological and psychological wellbeing. Understanding and addressing food insecurity not only as a nutritional concern but also as a pivotal factor influencing gut health and subsequent psychological parameters is essential for devising dietary interventions that promote overall wellness. Efforts toward enhancing food security may significantly mitigate the psychological toll associated with inadequate nutrition, offering potential pathways to improve quality of life for many patients.

## 7. Food Security as a Social Determinant of Mental Health

The broader implications of food insecurity extend beyond individual health to societal issues such as inequality. Food insecurity plays a role in perpetuating poverty cycles due to limited access to wholesome food, poor diet quality and related health problems, low productivity, and high mortality rates, which can amplify mental health challenges within these populations. In the study by Raskind in 2020, food insecurity was found to contribute to poverty cycles by intersecting with structural inequality, racism, and systemic oppression, leading to chronic stress, toxic trauma, and limited access to resources like affordable housing, childcare, and livable wages, thus perpetuating intergenerational cycles of disadvantage [75]. Dowler and O’Connor in 2012 found that even in developed countries like Ireland and the UK, food insecurity perpetuates poverty cycles by creating barriers for low-income households to access sufficient and healthy food. This demonstrates how the rights to food and health are interdependent [76]. In another study by Vilar-Compte et al., 2019, climate disasters, municipal-level poverty, and lower per capita GDP at the state level exacerbate food insecurity in Mexico, disproportionately affecting vulnerable populations and highlighting the significant influence of economic and environmental factors on access to nutritious food [77]. One such vulnerable demographic is women. In the study by Gepp et al. in 2022, flooding-induced food insecurity and subsequent mental health challenges for women in rural Bangladesh underscored the broader societal issues of vulnerability and inequality and the need for enhanced financial protection measures and livelihood adaptations to mitigate the impact of such climate-related events [78].

In developing nations, when households lack food security, it increases the likelihood of undernutrition, fueling a cycle of high morbidity and mortality, as highlighted by Khan and Bhutta in 2010, wherein factors like economic hardship, limited purchasing capacity, insufficient nutritional practices, and household food insecurity heighten the risk of undernutrition, perpetuating a damaging cycle where inadequate diet and disease burden exacerbate each other, significantly impacting children in regions like Asia, Africa, and Latin America [79].

Food insecurity also affects community mental health by straining social relationships, hindering productivity, and amplifying social tensions. Drawing from the study by Lombe et al., 2017, food insecurity is more likely to occur in households where there is a carer who suffers from a mental illness, as maternal mental health has a substantial impact on household food security. Having several mental health conditions and coming from a low-income household makes this susceptibility worse. Strong community ties, however, can serve as a protective factor, reducing both individual and group threats to food security [80].

Food insecurity, as a critical factor influencing mental health, requires inclusive interventions addressing socioeconomic gaps for fair access to nutritious food, aiding mental wellbeing and tackling broader societal challenges.

## 8. Sustainable Solutions and Global Efforts

The Sustainable Development Goals (SDGs) 2 and 3 intersect through their focus on nutrition, access to food, and overall wellbeing, highlighting the integral link between food security and mental health for achieving global Sustainable Development Goals.

The Sustainable Development Goal (SDG) 2 aims to end hunger, achieve food security, improve nutrition, and promote sustainable agriculture [81]. SDG 3 (Good Health and Wellbeing) aims to ensure healthy lives and promote wellbeing for all ages connected to food security and mental health [82]. Offering enough nourishing food combats malnutrition. This directly promotes mental health in several ways. Adequate food ensures the intake of essential nutrients such as vitamin B, iron, and polyphenols vital for brain function, positively impact mental health and cognitive abilities across various life stages, emphasizing the significance of nutrition in age-related cognitive decline prevention and management [83]. In the review by Spencer et al. from 2017, foods rich in essential nutrients like omega-3 fatty acids and polyphenolics found in fruits and vegetables were shown to play a critical role in brain function, cognitive abilities, and emotional wellbeing, impacting neurological conditions and cognitive deficits throughout life [84]. Gómez-Pinill in 2018 concluded that adequate food ensures individuals receive crucial nutrients vital for brain function, impacting mental health and cognitive abilities by influencing synaptic plasticity and neuronal resistance, as well as modulating cognitive processes through gut hormones and regulators like brain-derived neurotrophic factor [85].

Sustainable farming practices guarantee food supply, promoting mental health and communal stability resilience [86,87,88]. In the study by Elshaer et al., 2023, sustainable agriculture, specifically through farm-to-fork sourcing, positively impacted environmental sustainability through reducing the food supply chain’s carbon footprint and enhancing economic benefits for farmers and residents in Egypt [89]. In the study by Gebska et al. in 2020, farmers in Poland highlighted that greenhouse gas was reduced and water pollution was prevented due to sustainable agriculture [90]. In other instances, microbial bioeconomy has also contributed to sustainable agriculture. It has been used in energy recovery, waste management, recycling, biobased food and feed production [91].

Several innovative initiatives globally and locally target improved food security and mental health. Urban community gardens and farms offer accessible and locally grown produce, encourage community engagement, and utilize green spaces to enhance mental health, serving as nature-based solutions supporting sustainability and wellbeing [92]. A systematic review by Gregis et al. in 2021 found that community gardens offered health benefits across psychological, social, and physical dimensions, suggesting their potential as a viable urban public health promotion strategy. This emphasizes the need for comprehensive interdisciplinary impact assessments and locally based solutions for enhancing community wellbeing and environmental sustainability [93].

Mental health services such as stress reduction, therapy, and mindfulness practices are available on digital platforms and mobile apps. These resources are frequently coupled with nutrition information. A study by Oliveira et al. in 2021 showed that mobile mental health apps for college students exhibit strong acceptability, feasibility, and efficacy, suggesting their potential as valuable resources for university counselling services, particularly in addressing the challenges posed by the COVID-19 pandemic [94]. A more recent study by Diano et al. in 2023 highlighted how developing a safer mental health mobile application targeted emotion regulation skills within a transdiagnostic context [95].

The study by Barnes et al. in 2021 examined the effectiveness of strategies in improving the implementation of school-based policies, practices, or programs addressing child diet, physical activity, and obesity, revealing a variable range of effect sizes across studies. They found that methods for improving the nutritional value of food served in schools, implementing canteen policies, and scheduling physical education time are successful [96]. Such strategies for addressing deficiencies in medical nutrition education were discussed in a workshop study by Horn et al., 2019. They proposed a collaborative framework for enhancing competency-based nutrition curricula, interprofessional education, and research, thereby advancing nutrition training in health professional schools in the US and globally [97]. Various healthy eating programs and services like Infant Feeding Active play and NuTrition (INFNT), HEALing Matter, and the Victorian Aboriginal Community Controlled Health Organization (VACCHO) by the Victorian Government in Australia have contributed to improved nutrition and mental wellbeing [98].

To address these interconnected concerns, global advocacy efforts by organisations such as the United Nations and frameworks such as The Mental Health and Psychosocial Support Minimum Service Package (MHPSS) (for legislation that promotes mental health services, human rights, and food security) aim to make systemic reforms [99,100,101,102,103].

## 9. Call to Action

The policy framing dimension of food security is concerned with how the problem of food security is understood inside a government or governance framework, and it involves institutionalized norms and beliefs. Is food security, for example, defined largely as boosting agricultural production, or are social–economic, environmental, and health considerations also considered? The essential question for this dimension is whether the cross-cutting nature of food security is recognized, as well as the requirement for an integrative strategy [104]. Various frames may exist inside a polity, of course. A Department of Health, for example, will handle food security differently than a Department of Agriculture [105]. Some frames, however, may have greater resonance than others, particularly among high-level decision makers, and hence have a more substantial impact on the direction of policy interventions [106,107].

The following interventions and policies can be considered to address direct disruptions in the food system.

The subsidization of inputs can increase farmers’ use of fertilizers and improve seeds, pesticides, fuel, and machinery not constrained by labor shortages. The possibility of worker shortages becoming a recurring problem calls for additional labor supply interventions, such as creating “green corridors” (policies or initiatives facilitating migration or residency for specific reasons) for migrant workers [108]. Due to labor shortages, market closures, and changes in downstream processing and retail, entire harvests may perish before being available to consumers, resulting in a loss of food and income, price fluctuations, and food safety concerns, especially in the fresh produce sector. Post-harvest, storage, and processing interventions can reduce these losses in quality and quantity. Temperature-controlled supply chain solutions can considerably extend the shelf life of vegetables, thereby integrating food security [109,110,111]. In addition, home garden initiatives that promote nutritious traditional or biofortified crops and urban agriculture can potentially increase food availability in urban areas that are particularly vulnerable to food supply chain disruption [112].

Similarly, replacing suspended school feeding programs with take-home rations or cash transfers, as well as promoting and maintaining food fortification schemes, is critical to providing access to healthy meals and improving many people’s mental health [113]. Interventions targeted at directly boosting food access must reach vulnerable populations such as women and children, youth, the elderly, migrants, and impoverished informal-sector workers, as these groups are the most affected by food health issues [114]. Regional segmentation could help projects to be rolled out in appropriate locations or areas [115].

Food security can be quantified at the household, community, and national levels. At the national level, through policy making, the emphasis is on hunger and poverty caused by insufficient food consumption that makes it impossible to meet dietary energy requirements consistently. Direct experience perception-based surveys and diet quality assessments based on food intake are also employed to assess food security at the household, community or individual levels [116]. A community-based participatory intervention theory involving community people as collaborators can contribute to enlightening food security and also minimize health disparities on numerous levels [117].

Additionally, a variety of solutions have the potential to help solve the issues of food insecurity and restore mental health. One example is the creation of a visionary policy narrative that focuses on entire systems of initiatives (or a ‘solution ecosystem’) rather than individual programs [118,119]. Other strategies include aligning community-based initiatives with governmental goals and including stakeholders and the governments in decision making. Rather than the traditional top-down approach (identification of an agenda by researchers that may not reflect the community’s needs), developing a research project from the bottom-up (identification of critical issues for a specific population by community members) will more likely improve these populations’ participation and enthusiasm for the project and its intervention [120].

Overall, based on the above discussions, we highlight the following recommendations for integrating food security into mental health strategies.

- Implementing programs for vulnerable groups (women, children, elderly) with take-home rations, cash transfers, and fortified foods to boost food access and enhance mental wellbeing.
- Implementing policies related to food security that take mental health into account and vice versa.
- Providing resources for mental health initiatives within food security efforts.
- Encouraging cooperation between community organizations, food assistance programs, and mental health providers.
- Increasing public awareness of the adverse effects that food insecurity has on mental health by launching campaigns and educational programs.
- Investing in research to determine the efficacy of integrated approaches to better understanding mutually beneficial connections.
- Supporting efforts in urban agriculture that promote traditional and biofortified crops in high-risk urban regions to improve food availability, reduce supply chain interruptions, and improve mental health.

## 10. Conclusions

The impact of food insecurity on mental health spans beyond individual wellbeing, extending to societal issues such as poverty cycles and community mental health. Addressing this complex interplay necessitates comprehensive interventions integrating education, income, housing, social support, and healthcare access. Furthermore, the discussion underscores the interconnectedness between Sustainable Development Goal 2 (ending hunger) and Goal 3 (promoting good health), emphasizing the pivotal role of adequate nutrition in mental health and cognitive abilities. Strategies ranging from sustainable farming practices to urban agriculture initiatives and digital mental health services demonstrate avenues for enhancing food security and mental wellbeing. This highlights the need for collaborative interdisciplinary efforts and systemic reforms to address these interconnected challenges and foster community wellbeing and environmental sustainability.

This study is crucial as it delves into the intricate connections between food security and mental health, recognising their impact on overall wellbeing. By explicitly addressing the mental health repercussions of food insecurity, this narrative review contributes valuable insights for achieving Sustainable Development Goals related to hunger and health. The study’s emphasis on comprehensive interventions and the influence of socioeconomic factors underscores the urgency of targeted strategies. Understanding these interconnected factors is essential for policymakers, healthcare professionals, and communities. It guides them toward informed decisions and interventions that significantly enhance global wellbeing and contribute to broader societal goals.

As we navigate the intricate relationship between food security and mental health, what transformative steps can we take to ensure equitable access to nutritious food and bolster mental wellbeing for generations to come, fostering a world where both thrive hand in hand? The future presents a pivotal opportunity: the power to forge a healthier world lies in our commitment to interdisciplinary collaboration and systemic reforms that prioritize equitable access to nutritious food and mental health support, ensuring collective wellbeing and societal resilience.
